# Model-based estimation of heart movements using microwave Doppler radar sensor

**DOI:** 10.1186/s40101-024-00373-4

**Published:** 2024-10-21

**Authors:** Takashi Ota, Kosuke Okusa

**Affiliations:** 1 Department of Data Science for Business Innovation, Graduate School of Science and Engineering, Chuo University, Tokyo, 112-8551 Japan; 2 Department of Data Science for Business Innovation, Faculty of Science and Engineering, Chuo University, Tokyo, 112-8551 Japan

**Keywords:** Doppler radar, Heart rate, Heart movements, Heart radius, Electrocardiography, Mathematical model, Template matching method

## Abstract

**Background:**

Heart rate is one of the most crucial vital signs and can be measured remotely using microwave Doppler radar. As the distance between the body and the Doppler radar sensor increases, the output signal weakens, making it difficult to extract heartbeat waveforms. In this study, we propose a new template-matching method that addresses this issue by simulating Doppler radar signals. This method extracts the heartbeat waveform with higher accuracy while the participant is naturally sitting in a chair.

**Methods:**

An extended triangular wave model was created as a mathematical representation of cardiac physiology, taking into account heart movements. The Doppler radar output signal was then simulated based on this model to automatically obtain a template for one cycle. The validity of the proposed method was confirmed by calculating the PPIs using the template and comparing their accuracy to the R-R intervals (RRIs) of the electrocardiogram for five participants and by analyzing the signals of eight participants in their natural state using the mathematical model of heart movements. All measurements were conducted from a distance of 500 mm.

**Results:**

The correlation coefficients between the RRIs of the electrocardiogram and the PPIs using the proposed method were examined for five participants. The correlation coefficients were 0.93 without breathing and 0.70 with breathing. This demonstrates a higher correlation considering the long distance of 500 mm, and the fact that body movements were not specifically restricted, suggesting that the proposed method can successfully estimate RRI. The average correlation coefficients, calculated between the Doppler output signals and the templates for each of the eight participants, exceeded 0.95. Overall, the proposed method showed higher correlation coefficients than those reported in previous studies, indicating that our method performed well in extracting heartbeat waveforms.

**Conclusions:**

Our results indicate that the proposed method of remote heart monitoring using microwave Doppler radar demonstrates higher accuracy in estimating the RRI of the electrocardiogram while at rest sitting in a chair, and the ability to extract the heartbeat waveforms from the measured Doppler output signal, eliminating the need to create templates in advance as required by conventional template matching methods. This approach offers more flexibility in the measurement environment than conventional methods.

## Background

Vital signs are instrumental in the detection and monitoring of physical medical problems. One important means of observing vital signs [1–3] is monitoring the heart, commonly done by electrocardiography (ECG) [4–6], photoplethysmography (PPG) [7–12], sphygmomanometer [13–15], ultrasound [16–18], computed tomography (CT) [19–21], and magnetic resonance imaging (MRI) [22–24]. Although these heart monitoring procedures are provided during medical checkups such as routine physicals using dedicated equipment by professional staff at medical facilities, they may not be sufficient for early detection of diseases. This problem can be improved by routine self-checks of heart conditions at home [25, 26]. Among these means, PPG and sphygmomanometer are examples of heart monitoring systems [27] that are commonly used to monitor heart rate at home. In recent years, there are also wearable watch-type ECGs [27–29], but even slight body movements create noise. PPG measures heart rate by transmitting red or infrared light through the skin to detect blood volume changes in the microvascular bed tissue inside the fingers, wrists, and ears, but again the infrared light in the environment adds noise to the measurement. The digital sphygmomanometer [30] is the most popular home device because it can measure pulse rate and blood pressure simultaneously. It is an automated version of the conventional stethoscope method, and since they share similar detection principles, the measurement results are highly interchangeable [31, 32]. However, it also has a drawback in that the measurement results can vary depending on the way the cuff [32, 33] is worn. There are portable and home-use ECGs with a smaller number of electrodes [34, 35]. The quality of ECG results is affected by electrical noise such as changes in the surface impedance between the electrodes and the skin surface due to the state of electrode attachment, so there are challenges such as the way electrodes are attached to the body surface. The most convenient way to eliminate the problems associated with wearing these devices is remote heart monitoring. In this study, we developed a new method based on a mathematical model for remote estimation of the heart rate using a Doppler radar sensor. Conventional methods for remote heart rate measurement include laser [36–38] and radar [11, 12, 39–47]. The radar method cannot measure from as far away as the laser method, but it has advantages as a home monitoring system because it can penetrate non-metallic objects such as clothing and walls depending on its frequency. In some previous studies using radar methods, signals are transmitted toward the chest and the received signals are analyzed using various digital signal processing techniques [36, 43, 45, 47–52]. The main challenge of remote heart monitoring methods is eliminating the influences such as body movements or breathing, which make it difficult to detect the peaks of the heartbeat signal. Consequently, many studies have been carried out at close range, no more than 30 mm, under controlled conditions where the participants are lying in bed and holding their breath.

This paper describes a new remote heart monitoring method using a Doppler radar sensor. The key point of this study is that templates are generated through simulations of Doppler radar output signals based on a mathematical model of heart movements. While the conventional template matching method [36, 47, 53–55] provides higher accuracy in detecting peaks even with natural body movements and breathing, it requires significant effort. Our approach eliminates the need for prior signal measurements, unlike conventional template matching methods, and enables the generation of templates under various heart monitoring conditions through simulation. Therefore, the proposed method allows for heart monitoring from a greater distance compared to conventional methods, reducing the constraints of the measurement environment.

To validate the proposed method, the correlation between RRIs and PPIs of five participants, and the heart monitoring of eight participants were examined at rest while breathing and body movements were unrestricted. In this study, RRI refers to the interval between the peaks of the R wave in the QRS complex, which is a typical pattern based on the electrical activity of the heart. On the other hand, PPI refers to the interval between the peaks of the correlation coefficient curve calculated between simulated and measured heartbeat waveforms of the Doppler output signal, which reflects shape changes due to systole and diastole of the heart. Since both RRI and PPI parameters reflect the periodicity of cardiac activity, they are considered to be in good agreement. The novelty of the proposed method is demonstrated in the discussion, and finally, future insights into the method are provided.

## Materials

### Doppler radar sensor

A commercially available Doppler radar sensor (IPS-154, InnoCenT GmbH, Germany) was used for the 24 GHz radar measurements. In this study, a 24-GHz microwave radar was selected because of its high resolution for measuring heart movements from a certain distance [47, 53, 56–58]. The sensor has a normal output of 40 mW (maximum: 100 mW), a gain of 20 dB, and a full beam width (45° × 38°). After the signal output from the sensor was filtered and then amplified, the signal was sampled at a sampling interval of 1000 Hz by an A/D converter (CONTEC AI-16068AY-USB) and saved on a PC.

### Experimental setup and participants

**Fig. 1:**
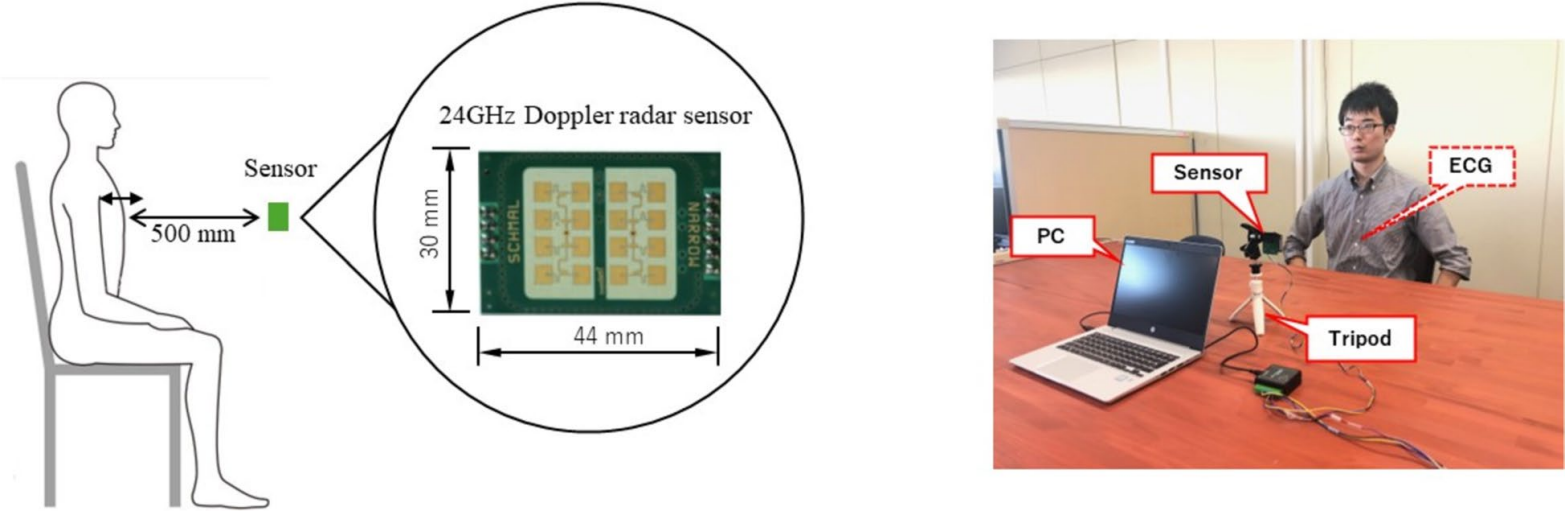
Schematics of experimental environment

The experimental setup is shown in Fig. 1. The Doppler radar sensor was fixed to a tripod set 500 mm away from the chest surface on the experimental table and focused on the underside of the sternum. Previous studies have not reported successful PPI measurements to estimate RRI, including Doppler sensor and other means, at distances greater than 30 mm [14, 15, 51, 59, 60]. We also used a wearable 2-lead electrocardiograph (myBeat, Union Tool Co.) as a reference [61–63], obtaining measurements with electrodes directly attached to the body at V1 and V2 positions of the precordial ECG. Healthy participants took part in each experiment, with all measurements being taken on the same day following a standardized procedure. The sensor height was adjusted to match each participant. The participants were seated in chairs, wearing their normal clothing, and were in a rested state during the measurements. The only exception was during a part of Experiment 1, where they were asked to hold their breath.

### Experimental protocol

#### Experiment 1

**Fig. 2:**
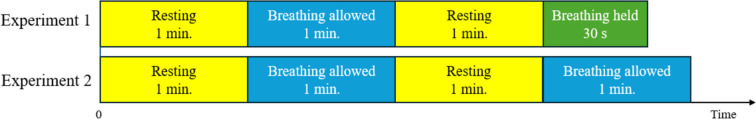
Experimental protocols of experiments 1 and 2

The participants were one female and four healthy male participants. Table 1 shows the physical characteristics of these five participants. Figure 2 depicts the experimental protocol. After a 1-min rest, the participants were seated in a chair and measured for 1 min at rest, with breathing allowed and their backs not touching the chair back. Then, they were measured again, but this time, the participants held their breath for 30 s to evaluate the effect associated with breathing. This set was repeated twice.

**Table 1:** Physical characteristics of the five participants

Experiment	Participant	Age	Sex	Height (cm)	Weight (kg)	BMI
1	A	22	M	171	53	18.13
	B	22	M	168	56	19.84
	C	24	M	174	63	20.81
	D	30	F	157	67	27.18
	E	58	M	174	68	22.46
	Average	31.20		168.80	61.40	21.68
	SD	13.72		6.31	5.95	3.09
2	F	22	F	154	51	21.50
	G	23	M	180	65	20.06
	H	23	F	155	44	18.31
	I	23	M	181	66	20.15
	J	22	M	172	70	23.66
	K	23	M	180	72	22.22
	L	22	M	177	77	24.58
	M	22	F	157	49	19.80
	Average	22.50		169.54	61.75	21.29
	SD	0.50		11.26	11.33	1.98

*M* Male, *F* Female, *SD* Standard deviation

#### Experiment 2

Experiment 2 aims to measure and analyze the *R* and *K* values, which represent heart radius and heart movement in the mathematical model. The participants were three females and five males, all healthy. The physical characteristics of the participants and the experimental protocol are shown in Table 1 and Fig. 2, respectively. Experiment 2 essentially followed the same conditions as Experiment 1, with the participants breathing at rest, their backs resting without holding onto a chair back, and measurements were repeated twice with a 1-min rest in between.

## Digital data processing

**Fig. 3:**
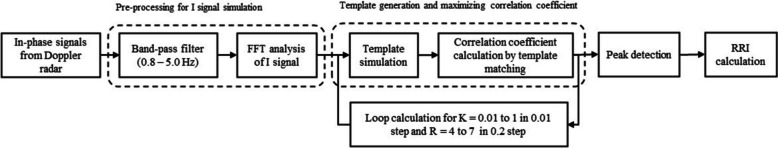
Schematic diagram of Doppler radar output signal processing and analysis

**Fig. 4:**
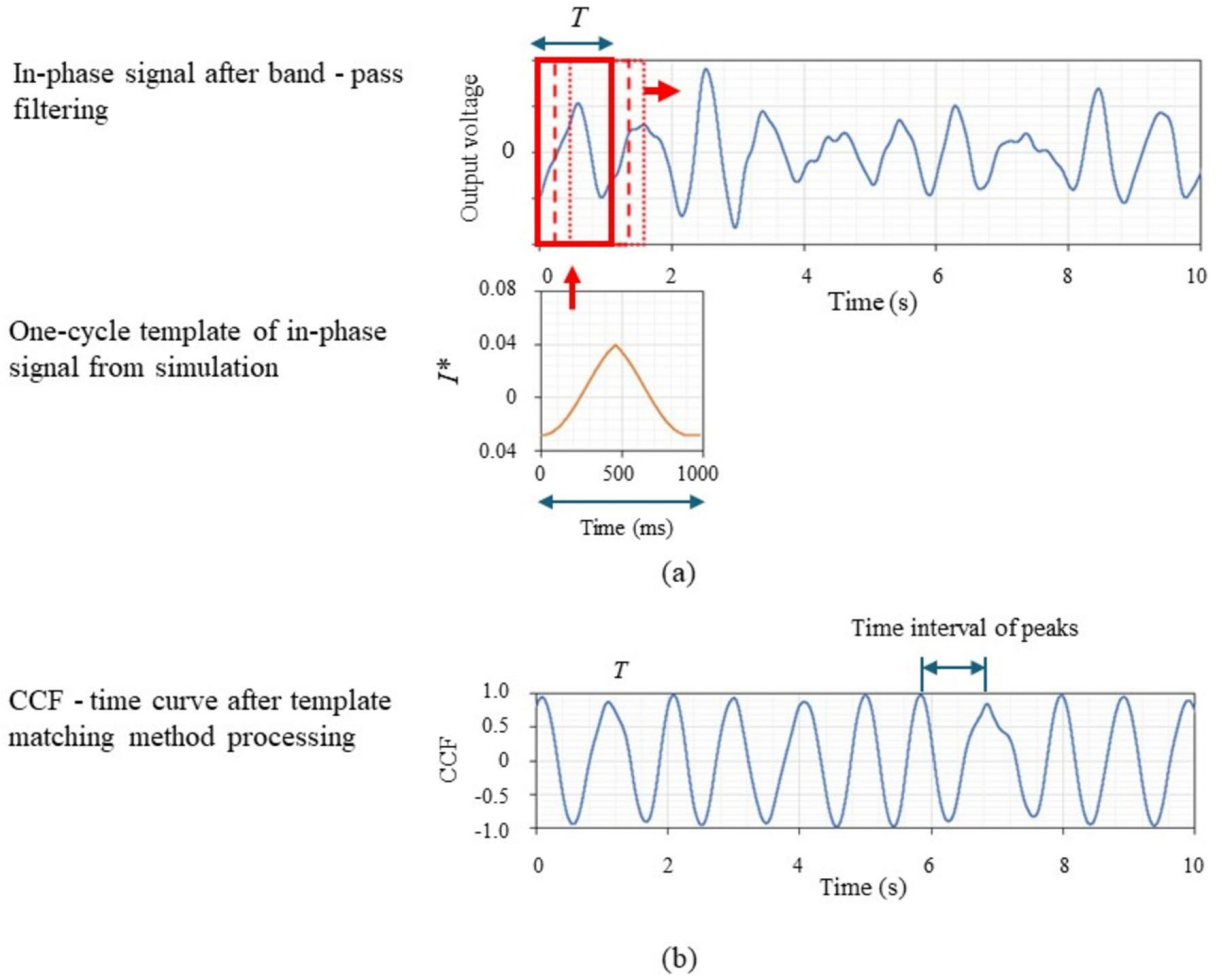
**a**, **b** Methodology of CCF calculation by template matching method

All signals were processed and analyzed offline according to the diagram shown in Fig. 3. The signal was bandpass filtered from 0.8 to 5 Hz, a setting that covers a range of 48 to 300 heartbeats per minute, to enhance the heartbeat waveform to a certain extent. After filtering, the FFT spectrum was checked, and the strongest peak was assumed to correspond to the heartbeat. The cycle of heart movements in the model was calculated from the inverse of the peak frequency, and one cycle of the output signal was calculated from a Doppler radar signal simulation using a mathematical model of heart movements and saved as template data. The correlation coefficient was calculated for each cycle, and the output signal was evaluated by setting a search window for the template and shifting the search window at the measurement time to calculate the correlation coefficient with the measured values over time as shown in Fig. 4a. Next, the median value of the peaks of the correlation coefficient curve was calculated, optimal *K* and *R* values were searched by maximizing the median value, and the peaks were detected using the phenofit library [64] of *R* (see Fig. 4b). Finally, the instantaneous PPI was estimated from each interval of the peaks over time. A PPI series, with the effect of respiratory sinus arrhythmia removed, was calculated by applying a low-pass FIR filter with a cut-off frequency of 0.05 Hz to eliminate respiratory variability [65, 66]. After the data processing described above, all data were saved on a PC. In this paper, only the in-phase signal from the I/Q modulator was used (Eq. (7)) among the output signals. The same estimation method can be used for analysis with the *Q* wave.

### Doppler radar signal simulation

The received signal is converted by an I/Q modulator, and its phase change can be used to calculate the change in distance between the sensor and the participant. The round-trip time, ${T}_{r}$, from when the sensor transmits microwaves to when it receives them, can be expressed as follows: where time is $t$, the speed of light is $c$, the distance of the object from the sensor is $D\left(t\right)$, and the velocity of the object is $v\left(t\right)$.

$${T}_{r}=\frac{2D\left(t\right)}{c}=\frac{2}{c}\left({l}_{0}+{\int }_{0}^{t}v\left(t\right)dt\right)$$

The signal received has a round-trip time delay, which results in a phase shift $\phi$. This phase shift can be calculated using the following equation, where ${f}_{0}$ represents the transmission frequency.

1 $$\phi \left(t\right)=2\pi {f}_{0}{T}_{r}\left(t\right)=\frac{4\pi f}{c}D\left(t\right)$$

Of the signals modulated by the I/Q modulator, high-frequency components are removed using a low-pass filter to obtain the following in-phase and quadrature signals. ${A}_{s}$ and ${A}_{r}$ are the amplitudes of the transmitted and received waves, respectively.

2 $$I\left(t\right)=\frac{{A}_{s}{A}_{r}}{2}\text{cos}\left(\phi \left(t\right)\right)$$

3 $$Q\left(t\right)=\frac{{A}_{s}{A}_{r}}{2}\text{sin}\left(\phi \left(t\right)\right)$$

**Fig. 5:**
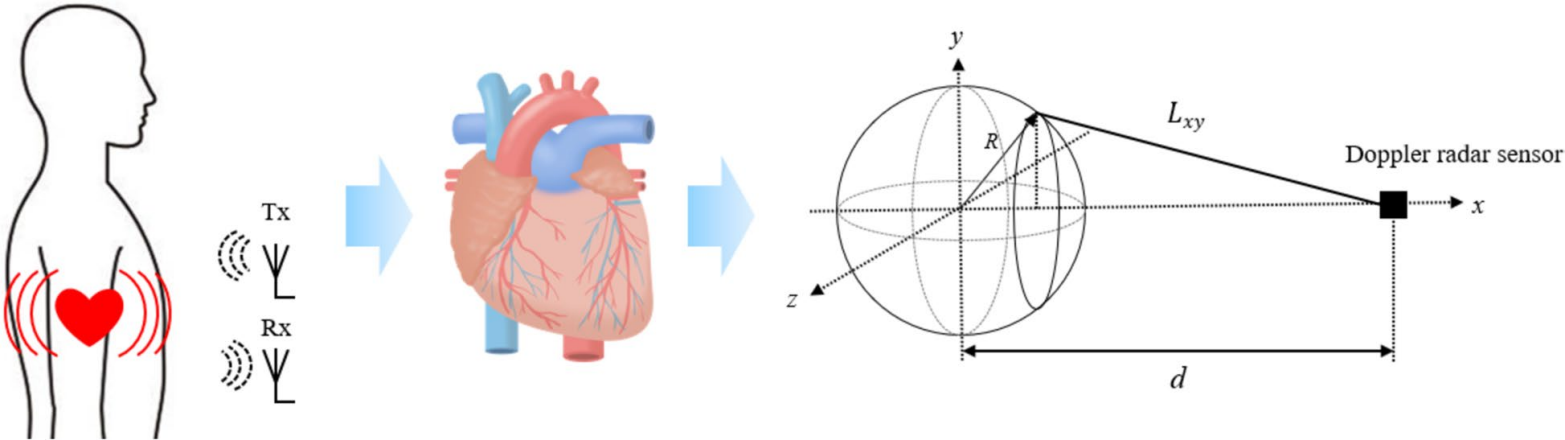
Schematic diagram of heart and illustration of location of heart and sensor

This indicates that the output signal of the Doppler radar sensor depends on $D\left(t\right)$. In this study, we modeled the heart to calculate the distance $D$ between the sensor and the chest surface of the participant. As depicted in Fig. 5, we assumed the heart to be a sphere with radius $R$. The distance $D$ was then calculated by integrating ${L}_{xy}$, which represents the distance between a point ($x$, $y$, $0$) on the $x$-$y$ plane and the sensor, across the plane.

4 $$D=2\pi \int {L}_{xy}dx=2\pi {\int }_{0}^{R}\sqrt{{(d-x)}^{2}+{y}^{2}}dx$$

Equation (4) is expanded by substituting Eq. (5), which represents the sphere, into Eq. (4).

5 $$\begin{array}{c}y=\sqrt{{R}^{2}-{x}^{2}}\\ D=2\pi {\int }_{0}^{R}\sqrt{{(d}^{2}-2dx+{x}^{2})+{(R}^{2}-{x}^{2})}dx=2\pi {\int }_{0}^{R}\sqrt{{d}^{2}-2dx+{R}^{2}}dx\\ =2\pi d{\int }_{0}^{R}\sqrt{1-\frac{2x}{d}+\frac{{R}^{2}}{{d}^{2}}}dx={\int }_{0}^{R}{\left[\left(\frac{-2}{d}\right)x+\left(1+\frac{{R}^{2}}{{d}^{2}}\right)\right]}^\frac{1}{2}dx\end{array}$$

Finally, an expression for the distance $D$ shown in Eq. (6) was obtained.

6 $$\begin{array}{c}D=2\pi d{\frac{2}{3\left(-\frac{2}{d}\right)}\left[{\left(\left(\frac{-2}{d}\right)x+\left(1+\frac{{R}^{2}}{{d}^{2}}\right)\right)}^\frac{3}{2}\right]}_{0}^{R}=\frac{-2\pi {d}^{2}}{3}{\left[{\left(\frac{-2dx+{d}^{2}+{R}^{2}}{{d}^{2}}\right)}^\frac{3}{2}\right]}_{0}^{R}\\ =\frac{-2\pi {d}^{2}}{3}{{\left(\frac{1}{{d}^{2}}\right)}^\frac{3}{2}\left[{\left(-2dx+{d}^{2}+{R}^{2}\right)}^\frac{3}{2}\right]}_{0}^{R}=\frac{-2\pi }{3d}{\left[{\left(-2dx+{d}^{2}+{R}^{2}\right)}^\frac{3}{2}\right]}_{0}^{R}\end{array}$$

The phase $\phi$ can be calculated from Eqs. (6) and (1). Also, the amplitude of the received waveforms, which are obtained from Eqs. (2) and (3), depends on the size of the object as seen from the sensor. Using the concept of solid angle $\omega$ to express this influence, Eqs. (2) and (3) were ultimately rewritten into the following equation. In this study, Eq. (7) was used to evaluate the in-phase signal received as Doppler output signal.

7 $$\begin{array}{c}{I}^{*}\left(t\right)=\omega \text{cos}\left(\phi \left(t\right)\right)\\ {Q}^{*}\left(t\right)=\omega \text{sin}\left(\phi \left(t\right)\right)\end{array}$$

### Mathematical model of heart movements

**Fig. 6:**
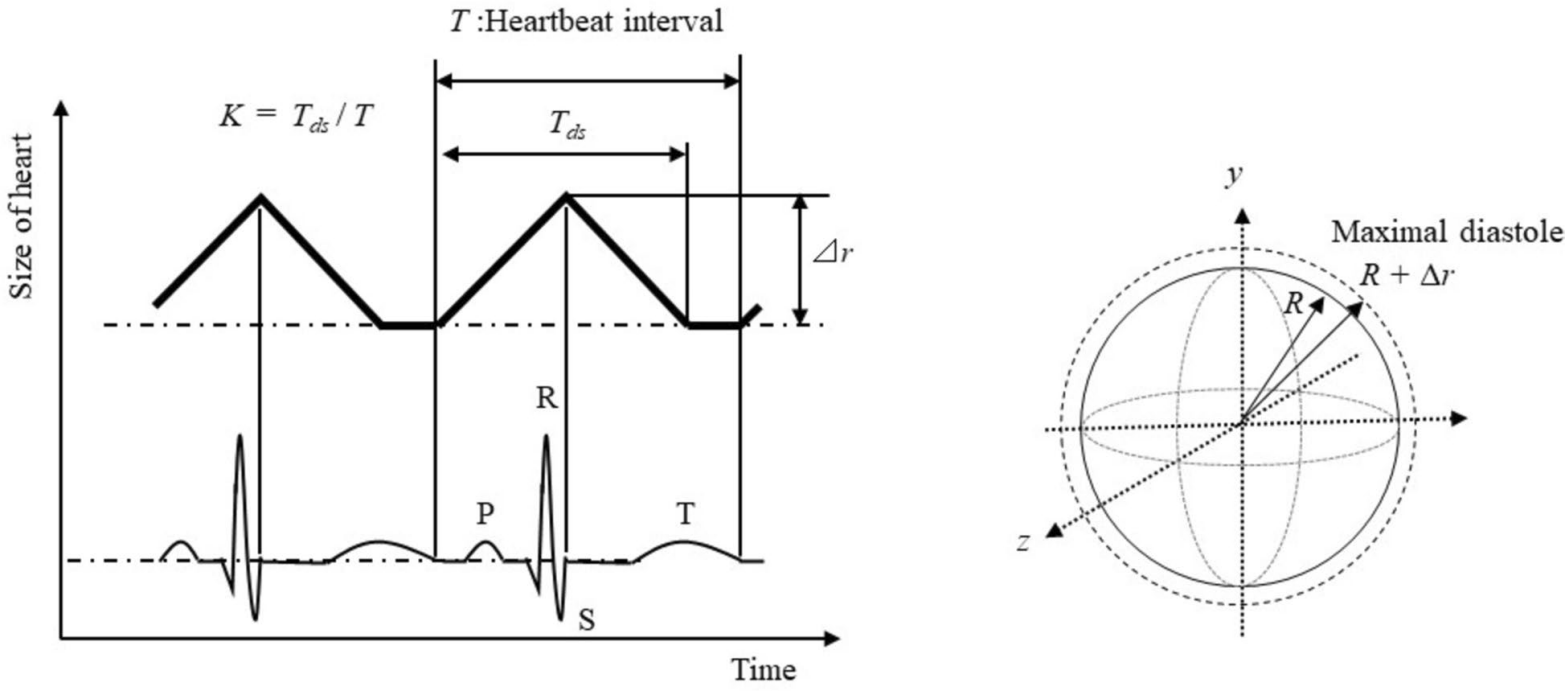
Schematics of extended triangular wave model in contrast to the ECG waveform

In cardiac physiology, the ventricular systole generally begins from around the top of the R wave to near the end of the *T* wave. As the aortic valve opens near the end of the *S* wave, the ventricular volume decreases between the *S* and *T* waves [67, 68]. The ventricular pressure peaks and then falls, causing the myocardium to contract at a slower rate near the start of the *T* wave. Ventricular diastole occurs with the opening of the tricuspid and mitral valves, and rapid diastolic filling starts following the systole. In this study, the extended triangular wave model is based on such cardiac physiology as shown in Fig. 6, where the endpoint of the triangular wave, in contrast to the ECG waveform [54, 69], is supposed to correspond to the *T* wave. The start point and the apex are assumed to be equal to the end of the *T* wave and *S* wave, respectively. In this model, a higher *K* value corresponds to an increased ratio of the triangular part, denoted as *T*_ds_ in Fig. 6. Similarly, a higher *R* value indicates a larger radius *R* of the sphere, which serves as a model of the heart, as shown in Fig. 6. In this paper, the amplitude ⊿*r*, which is illustrated in Fig. 6, is set to 10 mm, independent of the *R* value. On the other hand, it has been reported that a heartbeat causes minute movements of only 0.2 mm on the chest [59]. When the *K* value is 1, it represents a perfect triangular wave with cycle *T*. A *K* value greater than 0 and less than 1 represents the diastole-systole-stagnation characteristic. In this study, the analysis was conducted for *K* values ranging from 0.1 to 1 in 0.01 steps and *R* values ranging from 40 to 70 mm in 2 mm steps. Prior to the analysis described in this study, three mathematical models were evaluated for their predictive performance as heartbeat models. These models include a model assuming the heart to be a sphere, a large curvature sphere model assuming minute dilation and contraction of the body surface, and a one-dimensional model, known as the classical Doppler effect [70]. These models were used to simulate templates and to analyze the same signal. The sphere model produced the highest maximum median value of the peaks of correlation coefficients among the three models when the extended triangular wave model (see Fig. 6) was used. Therefore, only the sphere model as a heartbeat model was used to analyze output signals in this study.

**Fig. 7:**
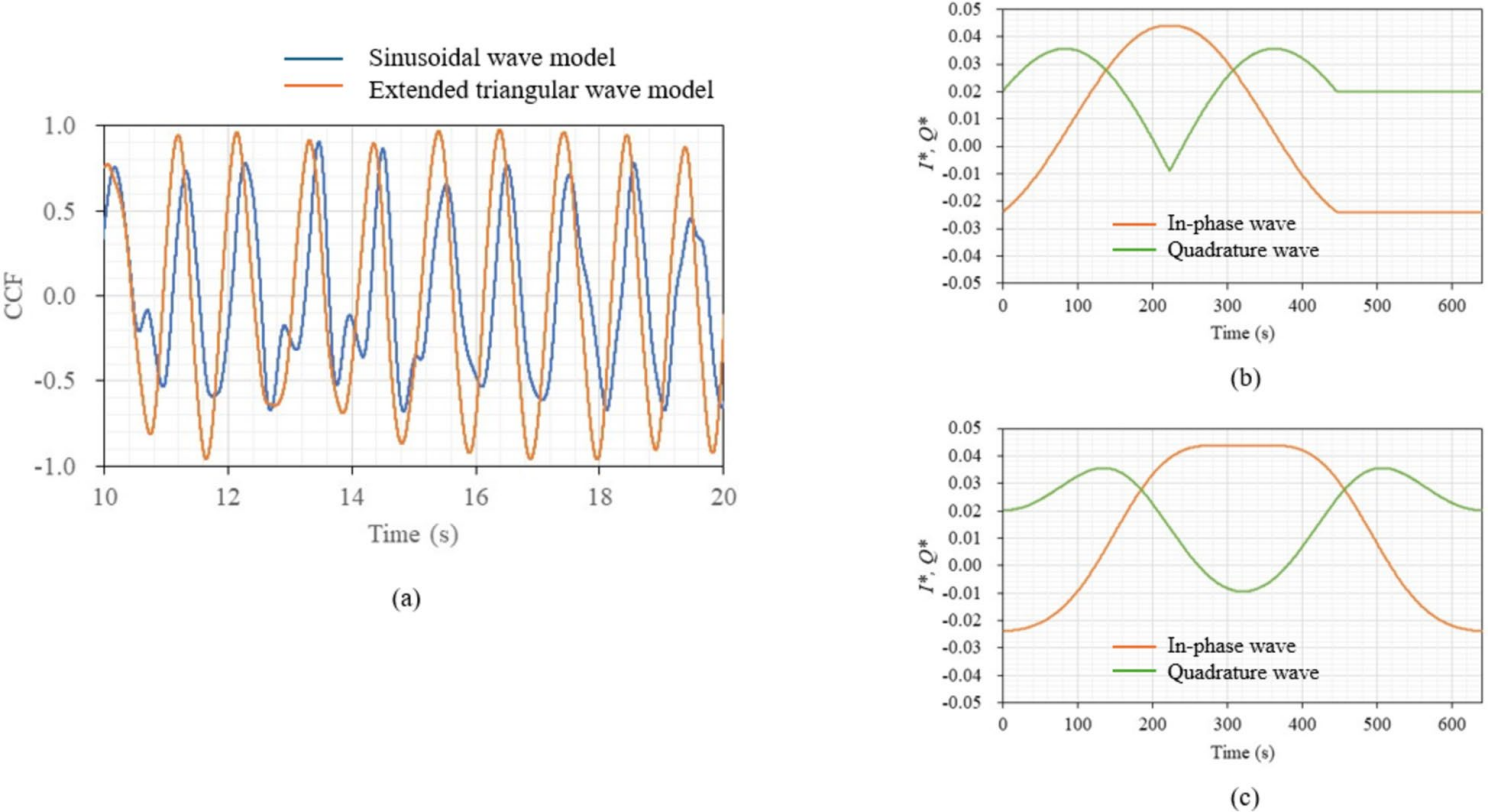
**a** Changes in correlation coefficients between simulations and output signal over time for extended triangular wave and sinusoidal models. **b** Simulated in-phase and quadrature waves by extended triangular wave model. **c** Simulated in-phase and quadrature waves by sinusoidal wave model

For comparison, the sinusoidal wave model, a standard for waveform analysis, was also used to simulate a template and to analyze the same signal. The results are shown in Fig. 7a, whereas Fig. 7b and c show the simulated templates by the extended triangular wave model and sinusoidal wave model, respectively. The maximum median value of the peaks of the correlation coefficient curve for the sinusoidal wave model was about 0.7, compared to 0.9 for the extended triangular wave model. This result indicates that the extended triangular wave model is appropriate as a heart movement model, and thus, the model was used throughout this study.

## Results

### Experiment 1: relationship between RRI and PPI

**Fig. 8:**
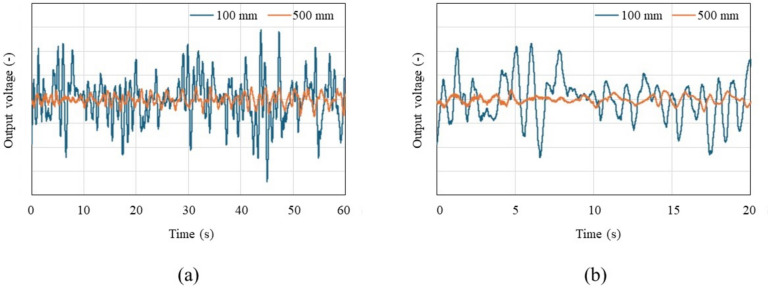
Examples of in-phase signals at rest when the distance between the body and the sensor is 10 cm and 50 cm, seated in a chair in a rested state with normal breathing, over a duration of 60 s (**a**). **b** Provides an enlarged view of a, focusing on a 20-s segment

**Fig. 9:**
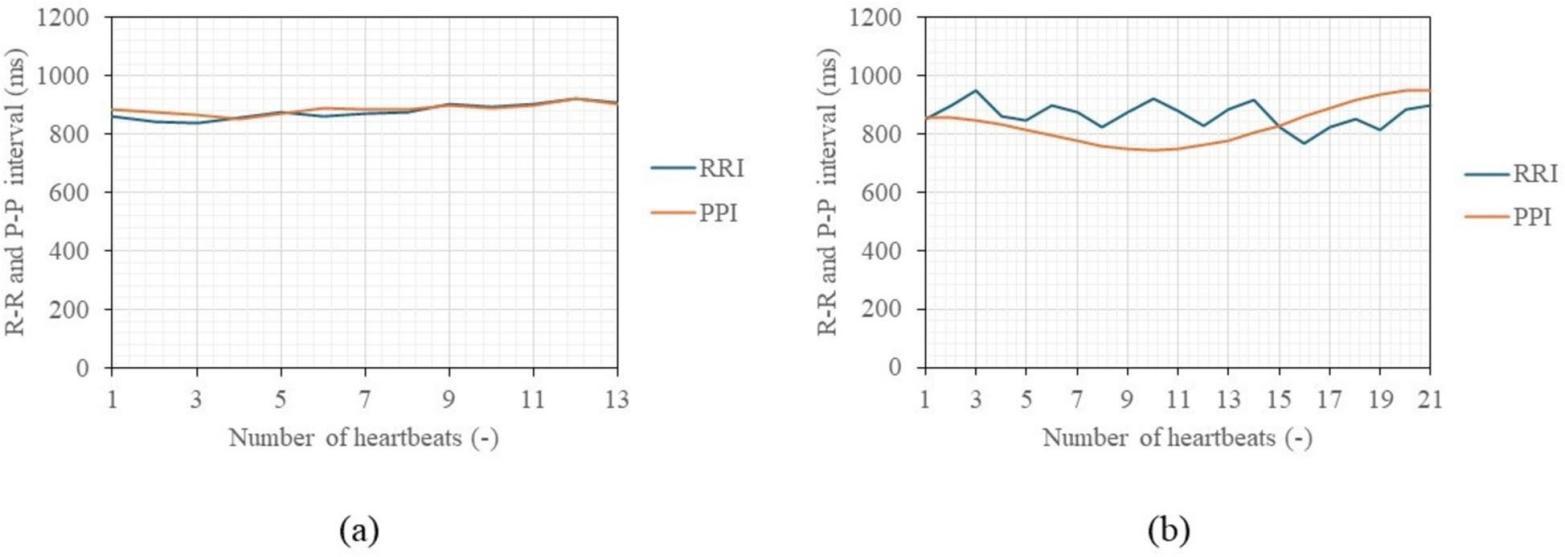
Example of comparison of R-R intervals and peak-to-peak intervals of participant A over the heartbeats when breathing was held (**a**) and allowed (**b**)

**Fig. 10:**
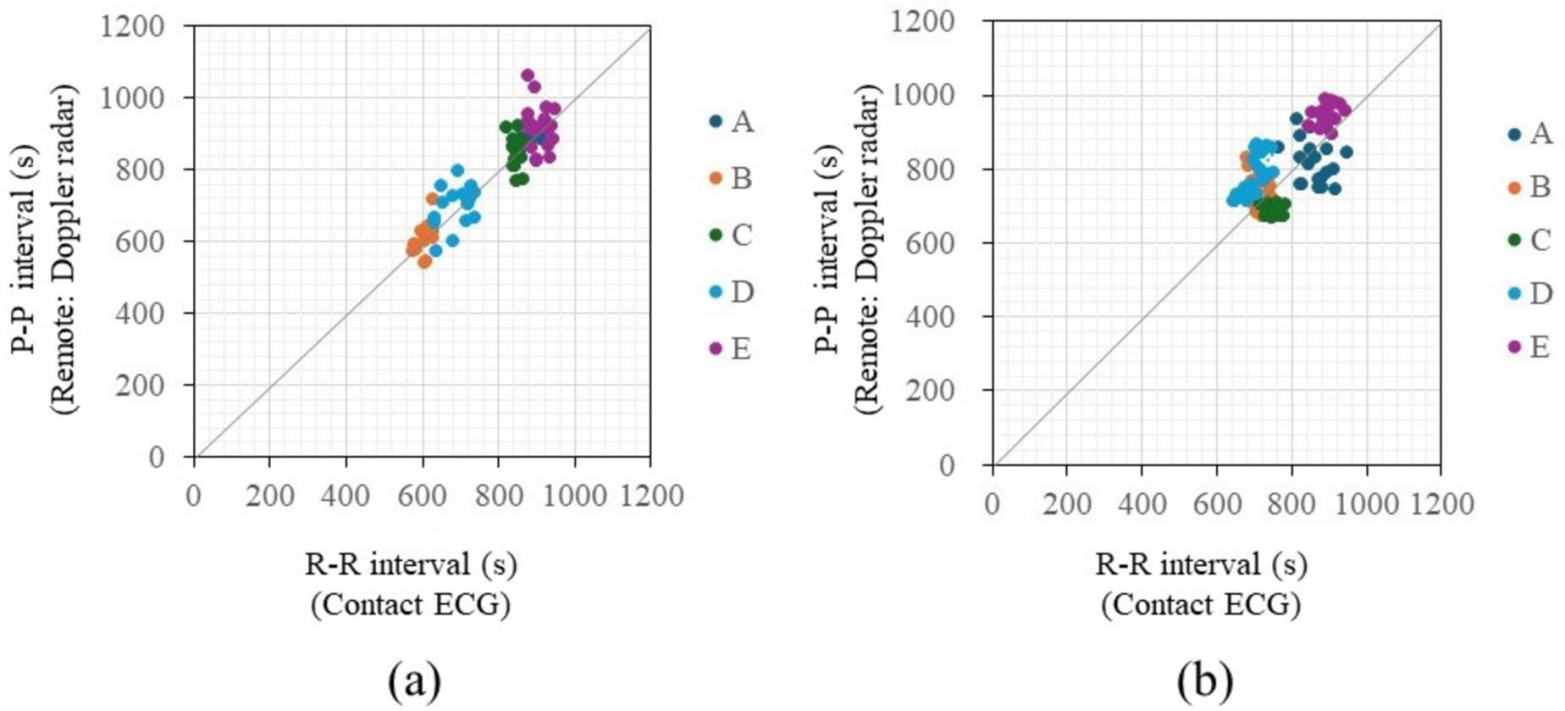
Comparison of the two results measured by the contact method as a reference and the estimation results based on remote measurement when breathing was held (**a**) and allowed (**b**)

**Fig. 11:**
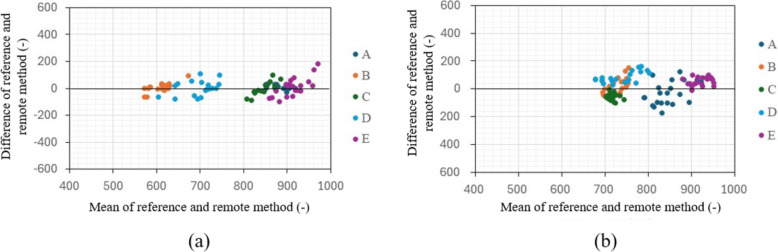
Bland-Altman plot for all participants when breathing was held (**a**) and allowed (**b**)

Figure 8a displays a graph of in-phase signals over 1 min when the distance between the chest surface and the sensor is 100 mm and 500 mm, with the subject seated shallowly in a chair in a rested state, allowing for breathing and body movements. Figure 8b presents an enlarged view of Fig. 8a, focusing on a 20-s segment. The vertical axis of the figures represents the normalized voltage value of the 16-bit output signal. A waveform with a heartbeat cycle of about 1 s appears to be superimposed on a visually long cycle amplitude, which seems to be due to respiratory harmonics or random body movements associated with breathing. The signal intensity decreases significantly as the distance between the chest surface and the Doppler sensor increases, indicating that it becomes more susceptible to the artifacts. The electrocardiograms and output signals of the Doppler radar for the five participants were measured simultaneously using electrocardiography and the proposed method to pair each heartbeat’s RRIs and PPIs when the participants held their breath and when they were breathing. Figure 9 displays a comparison between RRIs by ECG and PPIs over the number of heartbeats by the proposed method of participant A when breathing was held (a) and allowed (b). When breathing was involved, periodic variations in RRI were observed, a behavior seen in all participants. When breath was held, this variation was significantly smaller. The variations are attributed to respiratory sinus arrhythmia, which causes the RRI to fluctuate in synchrony with respiration. When breath was held, this variation was significantly reduced. Figure 10a and b illustrate the relationship between the RRIs and the PPIs for five participants when breathing was held and allowed, respectively. A strong correlation coefficient between the two was found when breath was held (*r* = 0.93, *p* < 0.001). The case where the participants were breathing showed a weaker correlation, but it was still moderate (*r* = 0.70, *P* < 0.001). Bland-Altman plots are presented in Fig. 11a and b. The vertical axis of the charts represents the difference between RRI and PPI for each heartbeat, whereas the horizontal axis displays the average of the RRI and PPI. The 95% limits of agreement, calculated from the graph results, were UCL = 15.13 ms and LCL = −8.22 ms (mean = 3.45 ms) when breath was held, and UCL = 23.65 ms and LCL = −4.52 ms (mean = 9.57 ms) when breathing was allowed. The results of the 95% limits of the agreement confirmed the same trend as the correlation coefficient in terms of a loss of accuracy when breathing.

### Experiment II: monitoring of heart

**Fig. 12:**
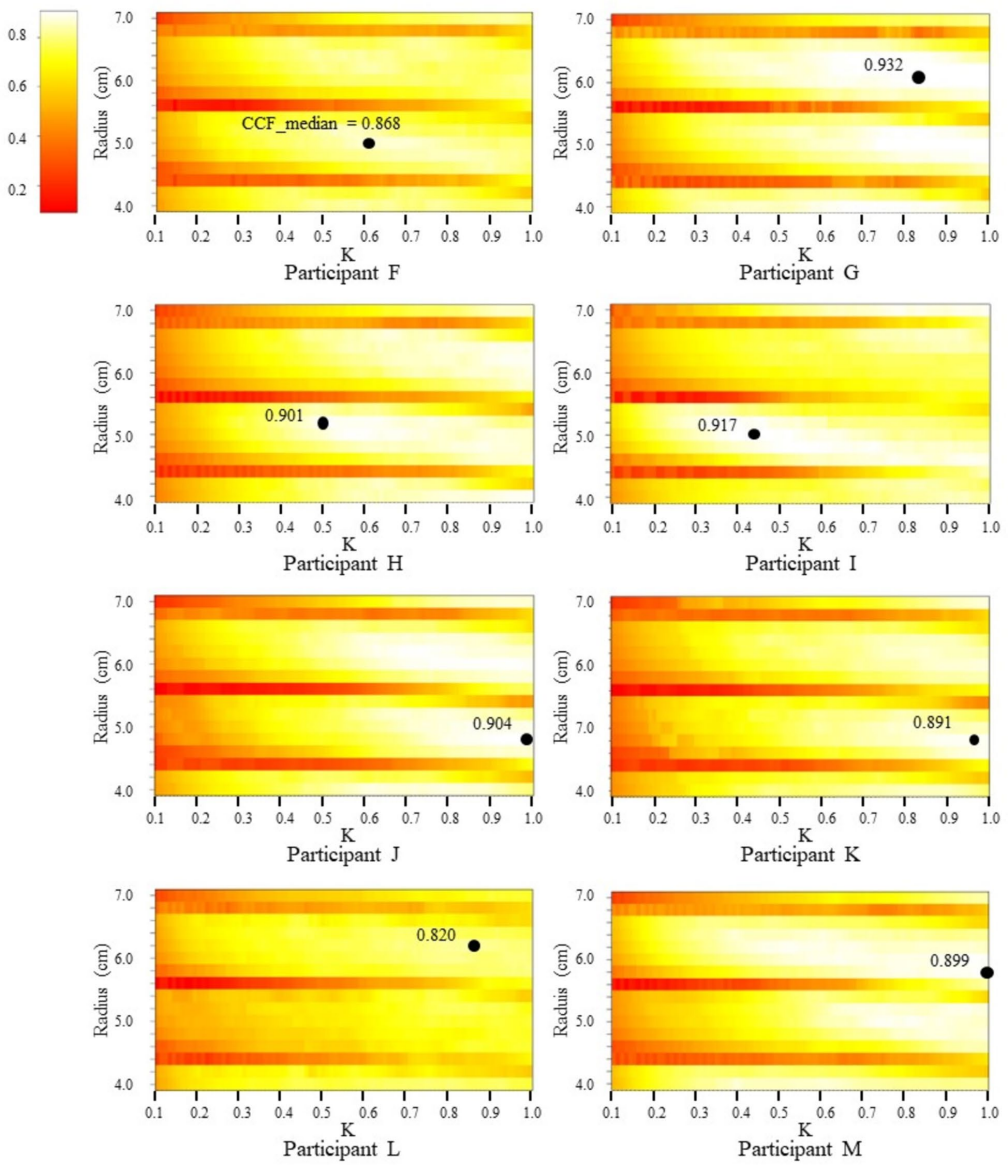
Heat map of CCF_median values (dots represent maximum CCF_median values)

Figure 12 displays heat maps of the median value of the peaks of the correlation coefficient (CCF_ median) calculated for each pair of *K* and *R* values in the Doppler output signal simulation of all participants. The maximum value of the CCF median was found through a grid search over the CCF median and is also depicted in the figure. To determine the maximum value of the CCF_median, the model parameters *K* and *R* were varied in the simulation. For each pair of *K* and *R* values, the CCF_median was calculated, which represents the median value of the peaks of the correlation coefficients between each template generated in the simulation and the Doppler output signal during the measurement period. This process was repeated for all combinations of *K* and *R*, resulting in a set of CCF_medians, each corresponding to a specific *K* and *R* combination. The maximum value of the CCF_median was identified as the highest CCF_median among all the *K* and *R* combinations, indicating the optimal *K* and *R* values that best match the Doppler output signal. It was inferred that the higher the CCF_median, the greater the similarity between the Doppler output signal and the simulated template. Overall, CCF_median tended to increase with an increasing *K* value. A periodic change in CCF_median was observed for the *R* value. This is due to the intensity of the output signal being influenced by both the distance to the sensor and the reflection area, as can be inferred from the equations mentioned in the previous section. For example, as the *R* value increases, the change in the intensity of the output signal decreases due to a decrease in round trip time and phase shift of the received signal. Conversely, the intensity increases due to an increase in the reflection area. Our simulations are unique in their ability to reflect such contrasting effects. As shown in Fig. 12, different participants obtained different pairs of *K* and *R* values for which CCF_median is maximum. Table 2 shows the average and standard deviation values of CCF_median, along with the corresponding *K* and *R* values, when measured twice consecutively with an interval of at least 1 min for each participant. Doppler output signals were analyzed sequentially in 3-s intervals without overlapping throughout the measurement period to account for the effect of respiratory sinus arrhythmia. The average of the calculated maximum CCF_medians for each participant exceeded 0.95, indicating a strong correlation.

**Table 2:** Estimation results of CCF_median value, *K* value, heart radius *R*, and the average and standard deviation values for measurements 1 and 2, along with the physical characteristics of the participants

Participant	Maximum CCF_median Average ± SD		*K* Average ± SD		*R* (cm) Average ± SD		Age	Sex	Height (cm)	Weight (kg)	HRA (bpm)
	1st	2nd	1st	2nd	1st	2nd					
F	0.975 ±0.032	0.984 ±0.019	0.819 ±0.130	0.873 ±0.120	5.7 ±0.62	5.5 ±0.67	22	F	154	51	78
G	0.980 ±0.025	0.977 ±0.029	0.898 ±0.116	0.816 ±0.195	5.8 ±0.59	5.7 ±0.7	23	M	180	65	84
H	0.982 ±0.005	0.992 ±0.004	0.839 ±0.071	0.990 ±0.083	5.4 ±0.61	5.7 ±0.61	23	F	155	44	91
I	0.982 ±0.015	0.982 ±0.015	0.925 ±0.091	0.846 ±0.120	5.6 ±0.67	5.7 ±0.61	23	M	181	66	93
J	0.978 ±0.024	0.983 ±0.015	0.827 ±0.144	0.978 ±0.160	5.9 ±0.56	5.6 ±0.71	22	M	172	70	95
K	0.974 ±0.022	0.958 ±0.037	0.825 ±0.108	0.692 ±0.110	5.7 ±0.69	5.5 ±0.70	23	M	180	72	68
L	0.970 ±0.020	0.975 ±0.026	0.847 ±0.119	0.937 ±0.058	5.7 ±0.65	5.4 ±0.72	22	M	177	77	90
M	0.983 ±0.012	0.969 ±0.027	0.805 ±0.118	0.862 ±0.110	5.4 ±0.60	5.7 ±0.74	22	F	157	49	76

Sex: *M* Male, *F* Female, *HRA* Heart rate average, *SD* Standard deviation

## Discussion

### Implications of experiment 1 results

Peak-to-peak interval (PPI) was estimated using the proposed method by Doppler radar for the five participants and compared with the beat-to-beat R-R interval (RRI) by electrocardiography. The difference between the PPI and the RRI was larger when the participants were breathing than when the participants held their breath, and a certain extent of the effect of respiration was observed [65, 66]. However, a moderate correlation was obtained which was comparable or better than the previous studies using Doppler radar, including studies conducted by detecting only the peaks of the signal (*r* = 0.884, *p* < 0.247) [60] or using the conventional template method (*r* = 0.769, *P* < 0.001) [71], despite the measurements from a closer distance such as 10 to 30 mm. Therefore, it can be considered that the proposed method could successfully estimate the RRI (or heart rate) of participants in remote conditions even from a distance of 500 mm. From the Bland-Altman plot, for the difference between RRI and PPI series, the mean value was 3.45 ms with a range of 95% limits of agreement of 23.35 ms when breathing was held, whereas those increased by 9.57 ms and 28.17 ms when breathing was allowed, respectively. This is considered to be due to the effect of respiration, as also shown in the correlation coefficient between PPI and RRI.

### Implications of experimental 2 results

The parameters *K* and *R* of the mathematical model of heart movements were calculated for each of the eight participants. The *K* value represents the simple cardiac expansion-contraction-stagnation characteristics of heart movements that could potentially be corresponding to electrocardiography and correlated with a wide range of studies on heart disease [69, 72, 73]. A very high similarity was obtained by the proposed method, with an average of 0.95 or higher for the median value of the peak values of the correlation coefficients. This suggests that the templates created by the simulation of the Doppler output signal based on the mathematical model of heart movements described in this paper can detect heartbeat waveforms when body movements are as small as those allowed in this experiment in a natural state. However, further studies on the validity of the parameters *K* and *R* of the model require verification using MRI and other methods in the future.

## Conclusion

This paper discusses a new method for remote heart monitoring that estimates the beat-to-beat R-R interval (RRI) of an electrocardiogram using a Doppler radar sensor, based on the mathematical model of heart movements. Our template matching method provides higher accuracy in remote heart monitoring while participants are in a natural state, such as sitting in a chair, offering more flexibility in the measurement environment than conventional methods. This is achieved by generating templates through the simulation of Doppler radar output signals based on the mathematical model of heart movements and leveraging the template matching method. This contrasts with many previous studies that were conducted at close range and under conditions where participants were lying in bed and holding their breath.

For remote heart monitoring, radar is extremely effective because it is transparent to non-metallic materials, can measure from a distance, and is less expensive. These features allow heart monitoring through clothing and bedding during sleep when body movements are minimal. Heart disease often manifests suddenly without prior symptoms. Continuous long-term measurement during sleep is expected to increase the likelihood of symptom detection.

While conventional studies of Doppler radar measurements utilize phase changes in the Doppler effect due to minute movements of the body surface caused by heartbeats, this study uses a mathematical model of heart movements. Because of this, our method has the potential for further extension to physiological considerations, allowing for the prediction of heart disease by taking into account various types of heart movements related to cardiovascular symptoms.

In this study, since the proposed method is still in the early stages of research, a grid search method is used to calculate the optimal values of *K* and *R* for evaluating the method. Although initial values do not need to be prepared, this approach results in longer calculation times. Reducing calculation time is a future challenge, and we believe that the use of time series analysis techniques, such as state space models, could improve the effectiveness of our heart monitoring method in practical applications.

## Data Availability

The datasets that support the findings of this study are available from the corresponding authors upon reasonable request.

## Abbreviations

RRI: Beat-to-beat R-R intervals of electrocardiogram
ECG: Electrocardiography
PPI: Peak-to-peak intervals of correlation coefficient curve
PPG: Photoplethysmography
CT: Computed tomography
MRI: Magnetic resonance imaging
UCL: Upper control limit
LCL: Lower control limit
CCF: Correlation coefficient
SD: Standard deviation
